# Identifying gene and protein mentions in text using conditional random fields

**DOI:** 10.1186/1471-2105-6-S1-S6

**Published:** 2005-05-24

**Authors:** Ryan McDonald, Fernando Pereira

**Affiliations:** 1 Department of Computer and Information Science, University of Pennsylvania, Levine Hall, 3330 Walnut Street, Philadelphia, Pennsylvania, USA, 19104

## Abstract

**Background:**

We present a model for tagging gene and protein mentions from text using the probabilistic sequence tagging framework of conditional random fields (CRFs). Conditional random fields model the probability *P*(**t**|**o**) of a tag sequence given an observation sequence directly, and have previously been employed successfully for other tagging tasks. The mechanics of CRFs and their relationship to maximum entropy are discussed in detail.

**Results:**

We employ a diverse feature set containing standard orthographic features combined with expert features in the form of gene and biological term lexicons to achieve a precision of 86.4% and recall of 78.7%. An analysis of the contribution of the various features of the model is provided.

## Background

Information extraction from biomedical text has attracted increasing research interest over the past few years. Several large scale annotated corpora have been developed [1] or are being developed [2] to facilitate this process. The first step in most information extraction systems is to identify the named entities that are relevant to the concepts, relations and events described in the text. In molecular biology, named entities related to genes, proteins or other biologically-active molecules are especially important.

Approaches to biological entity detection span a broad range from linguistic rule-based [3] to pure machine learning [4], as well as hybrids such as the system of Tanabe and Wilbur [5] that integrates an initial stochastic part-of-speech tagger to identify candidate genes using a special 'GENE' part-of-speech with a set of post-processing rules based on collected lexicons.

We present here a method for identifying gene and protein mentions in text with conditional random fields (CRFs) [6], which are discussed in the next section. Narayanaswamy *et al. *[3] suggest that rule-based systems make decisions on sets of textual indicator features and that these features may easily be exploited by supervised statistical approaches. Our method does just this by turning many of the post-processing steps of Tanabe and Wilbur [5] into features used in the extraction CRF. This is a single probabilistic tagging model with no application-specific pre- or post-processing steps or voting over multiple classifiers.

This makes the model quite general in that it may be extended to various other biological entities, provided appropriate lexicons are available.

The training, development and evaluation data for our system was provided by the organizers of BioCreative 2004 [7].

## Implementation

Presented here is an outline of conditional random fields and the implementation specifics of the model we use.

### Conditional random fields

**Figure 1:**
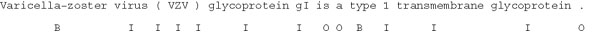
**Gene identification as a tagging problem. **A sample tagging of a sentence using the beginning, inside and outside tag labels. The sentence has two gene mentions, *Varicella-zoster virus (VZV) glycoprotein gI *and *type 1 transmembrane gylcoprotein*.

The identification of gene mentions in text can be implemented as a tagging task, in which each text token is labeled with a tag indicating whether the token begins (B), continues (I), or is outside (O) of a gene mention (see Figure 1).

Conditional random fields are probabilistic tagging models that give the conditional probability of a possible tag sequence **t **= *t*_1_, ... *t*_*n *_given the input token sequence **o **= *o*_1_, ..., *o*_*n*_:





In this definition, we assume that the *j*th input token is represented by a set *o*_*j *_of predicates that hold of the token or its neighborhood in the input sequence. The state *s*_*j*_(**t**) = (*t*_*j*-*k*+1_, ..., *t*_*j*_) encodes the tag *k*-gram ending at position *j*, for a suitable choice of *k*. We use *k *= 3 in this work, but some feature functions may ignore *t*_*j*-1 _or *t*_*j*-2 _and *t*_*j*-1 _to provide a form of back-off for rarely occurring tag trigrams or bigrams. Each *feature function f*_*i *_specifies an association between the predicates that hold at a position and the state for that position, and the *feature weight λ*_*i *_specifies whether that association should be favored or disfavored.

The most probable tag sequence for a given input sequence **o **can be obtained by applying a Viterbi-style algorithm [6,8] to the maximization



because *Z*(**o**) is constant for the given input.

The predicate set *o*_*j *_used to represent the *j*th input token picks out useful properties of the token and its context. For instance, if token *j *is the word "Kinase" and token *j *- 1 is the word "the", then *o*_*j *_might contain the predicates *WORD = kinase*, *WORD-1 = the*, and *WordIsCapitalized*, among others. The tag sequence uses the possible tags *B-GENE*, *I-GENE *and *O*, representing the beginning, inside and outside of a gene mention respectively. As noted before, each feature function relates input properties and a *k*-gram of tags. We use only binary features, for instance:



where tag_-*k*_((..., *t*_*j*-*k*_,...)) = *t*_*j*-*k*_.

The weight *λ*_*i *_for each feature should ideally be highly positive if the feature tends to be on for the correct labeling, highly negative if the feature tends to be off for the correct labeling, and around zero if the feature is uninformative. To achieve this, the model is trained so that the weights maximize the log-likelihood of the training data, :



To avoid degeneracy when some features are perfectly correlated and to reduce overfitting for rarely occurring features, we penalize the likelihood with a spherical Gaussian prior over feature weights [9]:



The variance hyper-parameter *σ*^2 ^determines the modeling trade-off between fitting exactly the observed feature frequencies and the squared norm of the weight vector. If we force the weights to be relatively small by choosing an appropriate small variance, the possibility of a single large weight dominating a decision is reduced. The value *σ *= 1.0 was chosen to maximize tagging accuracy on the development set. The log-likelihood function of conditional random fields, which generalizes the well-known case of logistic regression, is easily seen to be concave [6], as is the penalized likelihood (3). To maximize the penalized log-likelihood, we compute its partial derivatives with respect to the weights:



where  is the empirical feature count of feature *f*_*i *_and E[*f*_*i*_] is the model expectation of feature *f*_*i*_.

Maximizing concave functions has been well studied in numerical programming producing many iterative algorithms for finding the optimal weight settings. These include iterative scaling techniques [10] and gradient-based techniques [11,12]. All these methods require the calculation of the empirical and model expectations at each iteration.

The empirical expectations are trivially calculated by counting in the training data the number of times each feature occurs. The model expectations are given by:



Computing this sum directly is impractical because the number of possible tag sequences is exponential on training instance length. This is not a problem for maximum entropy models [13] because they model only single-label decisions so their expectations are just over the next label, not whole label sequences. However, the Markovian structure of the CRF model allows us to use an efficient dynamic programming algorithm to compute expectations over label sequences. First, we define a function that maps a single state at input position *j *to a set of allowed next states at position *j *+ 1, *T*_*j*_(*s*). To handle correctly the transitions at the start and end of a sequence, it is convenient to introduce a *start *state ⊥ and an *end *state. For instance, for the state *s *= (O, B-GENE):

*T*_*j*_(*s*) = {(B-GENE, I-GENE), (B-GENE, B-GENE), (B-GENE, O)}    1 ≤ *j *≤ |**o**|

*T*_0_(⊥) = {(⊥, O), (⊥, B-GENE)}

However *s*' = (O, O) ∉ *T*_*j*_(*s*) because *s *defines the current tag to be B-GENE, whereas *s*' defines the previous tag to be O, making it impossible for *s*' to follow *s*.

The expectation of a feature can then be computed as follows:



where *α*_*j*_(*s|***o**) is the unnormalized forward score of being in state *s *at position *j *given all observations before position *j *and *β*_*j*+1_(*s*|**o**) is the unnormalized backward score of being in state *s *at position *j *+ 1 given all the observations after position *j *+ 1. These values are calculated by the following recurrences:



where  iff *s *∈ *T*_*j*-1_(*s*'). The computation given by these recurrences is commonly called the forward-backward algorithm [8] and has *O*(*S*^2^*n*) running time, where *S *is the number of states and *n *the length of the sequence.

Having calculated the model expectations it is then possible to calculate the gradient of the objective function. This allows for the use of many gradient based optimization algorithms, the most simple of which is gradient ascent:



The gradient provides a search direction and a step size *η*, which can be chosen statically or be maximized dynamically by a line search. If chosen statically it must be sufficiently small to guarantee convergence. However, the smaller *η *is, the slower the algorithm will converge. Furthermore, gradient ascent does not take into account the curvature of the function, which also fundamentally slows its convergence speed. In order to consider the function's curvature, we require second order derivative information in the form of a Hessian. However, this matrix is far too large to invert (for use in Newton's method) or even store in memory, and the computation of individual elements is quite expensive [12].

Limited-memory quasi-Newton methods [14] approximate the Hessian by storing a history of update directions previously taken by the algorithm and combines them linearly with the current gradient to create the new search direction. These methods have been shown to be quite effective [11,12] for training log-linear models like CRFs. We use a history with four previous update directions, and train until the change in log-likelihood is sufficiently small.

CRFs require *O*(||*S*^2^*kn*) training time, and *O*(*S*^2^*n*) decoding time, where *k *is the number of training iterations required for convergence, *n *is the length of a observation sequence, *S *the number of states, and || the size of the training set [6].

We used the MALLET [15] implementation of CRFs and limited-memory quasi-Newton training. In addition, we relied on MALLET's *feature induction *capability [16], which is discussed in the next section.

### Relationship with maximum entropy

Conditional random fields are closely related to other conditional formalisms in the literature. The strongest connection is between CRFs and maximum entropy classification models [13]. Maximum entropy models and conditional random fields as well as other conditional models have become increasingly employed for tagging and shallow parsing due in large part to their ability to incorporate millions of rich and highly dependant features, unlike generative tagging approaches such as hidden Markov models [8], which can only handle sets of features that are independent of each other.

Maximum entropy models give the conditional probability of a class given an observation by:



To apply maximum entropy classification to tagging, we see the problem as a sequence of probabilistic decisions in which tag *t*_*j *_is chosen depending on previous tags and the input sequence. In the notation of the previous section, we would have:





Such a model is trained by taking each tag occurrence and its conditioning context in the training data as a separate training instance [17]. This training process is simpler than that for CRFs as it does not require forward-backward computations. For testing, a Viterbi-like algorithm finds the tagging sequence that maximizes (5).

While these models are easy to train, they are independently normalized at each position *j *(equation 4). That is, for each *j*, *P *(*t*_*j*_|...) has its own separate Σ_*t *_exp Σ_*i *_*λ*_*i*_*f*_*i*_((...),*o*_*j*_) normalizing expression in equation 4's denominator. In contrast, conditional random fields have a single combined normalizing denominator of *Z*(**o**) for the entire tag sequence **t **(equations 1 and 2). This independent normalization prevents decisions at different positions from being weighed against each other. This *label bias *problem [6] motivated the development of CRFs, and has been shown to adversely affect accuracy in realistic tagging tasks [12].

It might be argued that the lower accuracy of maximum entropy taggers is compensated by their faster training, which allows more complex models to be considered, for instance models with higher Markov order or more feature types. Indeed, tagging methods based on locally trained classifiers [18,19] need to exploit more context to alleviate label bias. However, any changes to a maximum entropy tagging model can be trivially applied to the corresponding CRF model, including changes in model order and feature choice. For all the tagging tasks that we have attempted, CRF models can be trained in under 20 hours (and in most cases under 10 hours), which is quite practical since training is done only a few times. This seems a small cost to pay for significant accuracy increases, such as a 10% relative reduction in error rate [12]. Applying the trained model uses exactly the same Viterbi algorithm for CRFs as for maximum-entropy classification. It would be useful to compare directly the two approaches for gene and protein identification, although there are already results for similar tagging tasks [12]. However, our experiments rely on feature induction (described later), so the comparison would need extending feature induction to maximum entropy tagging, which is conceptually straightfoward but would require a significant implementation effort.

### Feature set

Feature-based models like CRFs are attractive because they reduce each problem to that of finding a feature set that adequately represents the task at hand. We used features based on word as well as orthographic predicates, shown in Table 1, as well as character-*n*-gram predicates for 2 ≤ *n *≤ 4. These predicates help the system recognize informative substrings (e.g. 'homeo' or 'ase') in words that were not seen in training. We also included word prefix and suffix predicates, also of lengths 2 ≤ *n *≤ 4. This may seem redundant, but prefix and suffix predicates also take into account the position of the *n*-gram in the word, which can often be informative. For example *ase *occurring at the end of a word is much more informative then just knowing *ase *is contained in the word (i.e *laser *vs. *kinase*). We include predicates that indicate whether the current token occurs within brackets or inside quotations. Finally, we used {-1, 0, +1} as our predicate window, meaning that for token *j *we included all predicates about tokens *j*-1 and *j*+1 as well as all predicates for token *j*.

Even with this very simple set of predicate-based features, performance on the development data was reasonable (see Table 2 row A). In order to add expert knowledge to the model, we focused our attention on the gene and protein tagger *ABGene *[5]. ABGene is a hybrid model that uses a statistical part-of-speech tagger to identify candidate genes by labeling them with a special part-of-speech 'GENE'. Once the candidate genes are found a series of post processing rules are initiated to improve the results. Specifically, ABGene uses a set of lexicons to remove false positives and recover false negatives. These include general biological terms, amino acids, restriction enzymes, cell lines, organism names and non-biological terms meant to identify tokens that have been mislabeled as 'GENE'. To recover false negatives, ABGene utilizes large gene lexicons coupled with context lists to identify possible mentions. Another post-processing step identifies tokens that contain low frequency trigrams, compiled from MEDLINE, to identify possible gene candidates, since gene and proteins names often contain unusual character trigrams.

A straightforward method of integrating these post-processing steps into our model is to create predicates indicating whether a token occurs in one of the ABGene lexicons. For multi-token entries, we required that all tokens of the entry were matched. We used the window {-1, 0, +1} for these predicates too. Table 2 rows C through F summarizes the effect of adding these lexicons to the system. Rows C through F assume the use of feature induction, which is explored in the next section.

### Feature induction

So far we have only described features over a single predicate. Often it is useful to create features based on the conjunction of several predicates. For instance, the following feature would be useful:



This is because the token p-53 can either be in a gene mention (when it is followed by the word 'mutant') or be in a mutation mention (when it is followed by the word 'mutations'). Hence the following feature would also be useful:



The system already has tens of thousands of singleton features, making it infeasible to create all such conjunctions. Even if it were computationally feasible to train a model with all conjunctions, it would be difficult to gather sufficient statistics on them since most conjunctions occur rarely if ever.

To solve this problem, McCallum [16] describes an implementation of feature induction for CRFs that automatically creates a set of useful features and feature conjunctions. The method scores candidate features *f *with their log-likelihood gain:



where *F *is the current set of model features,  the log-likelihood of the data using feature set *F *and  the log-likelihood of the data with the model extended with feature *f*.

Feature induction works by iteratively considering sets of candidate singleton and conjunction features that are created from the initially defined set of singleton features as well as the set of current model features. Only those candidates causing the highest gain are included into the current set of model features. Intuitively, features causing high gain provide strong evidence for many decisions. Thus, feature induction tends to discard infrequent features or non-discriminating features since, independently, their overall effect on the likelihood of the entire training set is usually marginal. There are serious computation issues with feature induction, primarily due to the fact that for each iteration and for each feature considered, the normalization term for every training sequence must be recomputed. However, the problem can be somewhat alleviated by making certain independence assumptions on the parameters as well as only including statistics on positions in the sequence that are mislabeled by the current parameter settings. McCallum [16] describes the procedure in more detail.

Table 2 rows A and B show the difference in performance using feature induction instead of just the predefined singleton features.

## Results

Our system was initially trained on 7500 annotated MEDLINE sentences with a development set of 2500 sentences. Training with feature induction took approximately 15 hours, which is substantially longer than training without feature induction. Once trained, the system can annotate sentences in less than a second. In terms of labor, the system took only a few days to build. This was primarily due to the availability of MALLET [15], which includes effcient implementations of both conditional random fields and feature induction. Once the basic tagger was implemented, the remaining effort focused on testing various spelling, contextual and lexicon features on the development data to improve performance.

For evaluation, we added the development set to the training data and evaluated on 5000 new unannotated sentences. The results are shown in Table 3. Entities were correctly identified by the system if and only if all and only the tokens of the entity were correctly detected.

## Discussion

Adding the ABGene lexicons made a significant improvement to both precision and recall. This is a very good indicator that additional domain knowledge may help to further improve the accuracy of the system. To determine which lexicons gave the best performance, we conducted experiments examining the effect of adding each type of lexicon individually to the model and tested the model on the development data. These results are outlined in Table 2. Each list made a small improvement to the overall accuracy of the system, with the gene lexicon contributing the largest improvement. Table 2 also shows the performance of the system without lexicons and feature induction.

An examination of system errors on the development data shows that a primary source of error came from properly labeled mentions that are off by one or more tokens. If the score is relaxed so that tagged entities are considered true positives if and only if one or more tokens overlap with a correct entry, then performance on the development data increases 7.5% absolute from 79.9% to 87.4% F1 measure. For an extreme example, consider the string *interleukin-1 [IL-1], tumor necrosis factor-alpha [TNF-alpha]*, which the tagger incorrectly returns as being one entity. The gold standard identifies 4 different entities within this string, *interleukin-1*, *IL-1*, *tumor necrosis factor-alpha *and *TNF-alpha*. The net result is that recall is penalized four times and precision is penalized once due to the four false negatives and one false positive. It appears that it is relatively easy to find pieces of text mentioning genes, but much harder to determine the exact boundaries of that mention. This hurts the system performance significantly since the scoring metric requires exact matches. However, entity tagging primarily exists to give some structure to text for higher level information extraction systems such as relation detection, fact generation and question answering. For these problems, having an entity correctly identified with improper spans can potentially still be useful.

## Conclusion

Overall, our experiments show that CRF models with carefully designed features can identify gene and protein mentions with fairly high accuracy even without features containing domain specific knowledge. However, such features, which in our case take the form of lexicon membership, can lead to improved system performance.

## Figures and Tables

**Table 1:** Orthographic features.

Orthographic Feature	Reg. Exp.
Init Caps	[A-Z].*
Init Caps Alpha	[A-Z] [a-z]*
All Caps	[A-Z]+
Caps Mix	[A-Za-z]+
Has Digit	.*[0-9].*
Single Digit	[0-9]
Double Digit	[0-9][0-9]
Natural Number	[0-9]+
Real Number	[-0-9]+ [.,]+[0-9].,]+
Alpha-Num	[A-Za-z0-9]+
Roman	[ivxdlcm]+ or [IVXDLCM]+
Has Dash	.*-.*
Init Dash	-.*
End Dash	.*-
Punctuation	[,.;:?!-+'"']

This defines the complete set of orthographic predicate used by the system. The observation list for each token will include a predicate for every regular expression that token matches.

**Table 2:** Effect of system components on development data

System	Precision	Recall	F-Measure
A. No Lex, No Feat. Ind.	0.793	0.731	0.761
B. No Lexicons	0.807	0.744	0.774
C. Trigrams	0.811	0.759	0.784
D. Non-gene Lexicons	0.818	0.743	0.778
E. Gene Lexicons	0.812	0.775	0.793
F. All Lexicons	0.817	0.782	0.799

**A) **System containing no lexicon features and does not use feature induction. **B) **Same as A, except feature induction is used. **C) **Same as B, except features using the infrequent trigram lexicon are used. **D) **Same as B, except features using the non-gene lexicons are used. **E) **Same as B, except features using the gene lexicon are used. **F) **Same as B, except features using all lexicons are used.

**Table 3:** Precision and recall numbers for the system on the unseen evaluation data

System	Precision	Recall	F-Measure
No Lexicons	0.830	0.773	0.801
Lexicons	0.864	0.787	0.824

Precision and recall numbers for the system on the unseen evaluation data. Precision is measured by the fraction of predicted gene mentions that are correct and recall by the fraction of actual gene mentions that were identified. Two system results are provided. The first is for the system that contains only features extracted from the training data. These results are presented in the row *No Lexicons*. The second set of results are for the system that also contains features extracted from external lexicons. These results are presented in the row *Lexicons*.
